# Utilization of a public subsidy scheme for dental care services among socially vulnerable citizens out of labor in Copenhagen, Denmark

**DOI:** 10.1080/00016357.2023.2279606

**Published:** 2024-03-26

**Authors:** Marie Skov Kristensen, Annette Kjær Ersbøll, Ingelise Andersen, Esben Boeskov Øzhayat

**Affiliations:** a Department of Odontology, Faculty of Health and Medical Sciences, University of Copenhagen, Copenhagen, Denmark; b National Institute of Public Health, University of Southern Denmark, Copenhagen, Denmark; c Department of Public Health, Section of Social Medicine, Faculty of Health and Medical Sciences, University of Copenhagen, Copenhagen, Denmark

**Keywords:** Dental care delivery, low-income population, out of labor, social inequalities, social vulnerability

## Abstract

**Objective:**

Addressing social inequality in oral health and access to dental care is a global concern. This study aims to describe the utilization of a public subsidy scheme targeting vulnerable individuals out of labor in Copenhagen municipality (2013–2018) and to identify key characteristics of individuals eligible to apply.

**Material and Methods:**

Data from Copenhagen municipality were combined with data from population and health registers. Employing logistic regression analyses, we examined the association between demographic, socioeconomic, and health-related characteristics and (1) having applied, (2) being granted, and (3) using the subsidy.

**Results:**

The study included 65,174 individuals aged 18–65. Of these 10,369 (15.9%) applied for subsidies, submitting a total of 18,529 applications. Overall, 83% of the applications were granted and 85% were used. Significantly increased odds of applying for subsidies were observed among individuals receiving social benefits non-stop over the past year versus none (odds ratio [OR] = 15.45, 95% confidence interval [CI] = 14.24–16.76), aged 50–65 versus 18–29 years (OR = 4.41, CI = 4.15–4.69), and having combined indicators of social vulnerability versus none (OR = 2.90, CI = 2.73–3.07).

**Conclusions:**

While the utilization of the public subsidy scheme is low, individuals who apply are likely to be granted a subsidy and use it. Vulnerability was associated with greater utilization of the scheme, yet a substantial portion of those at risk of poor oral health did not take advantage of it.

## Introduction

Oral health has significantly improved across the Nordic countries over the past four decades. Nevertheless, the persistent social inequality in oral health remains a major concern [1]. Extensive evidence supports the association between low socioeconomic position and poor oral health [2–5], largely attributed to limited access to and underutilization of dental care services among vulnerable populations [6]. Hence, the ‘inverse care law’ [7] rules, saying that the most vulnerable and marginalized individuals in most need of dental care receive the least. Consequently, this underserved population faces an increased risk of advanced oral health effects [8,9], reduced quality of life [10], and social consequences affecting everyday life and employment [11]. Internationally, there is no consensus on a unified approach to improving access to dental care services for low-income and vulnerable individuals. However, user payment is cited as the primary barrier to accessing oral health care [12,13]. The World Health Organization (WHO) recommends, in alignment with the Danish Health Authority to reduce user payment for dental care services to minimize inequalities in oral health [14,15]. In Denmark, individuals who are out of labor and receive social benefits can apply for a public subsidy for dental care services according to the Danish Active Social Policy Act. This subsidy is administered individually by municipalities across the country [16,17]. Nonetheless, the Danish Dental Association has reported a low uptake of the public subsidy scheme at a national level in 2017 [18]. Yet, no studies have described the utilization of the public subsidy scheme at an individual level and over time. Hence, this study aims to provide a comprehensive description of the utilization of the public subsidy scheme for dental care services in Copenhagen municipality from 2013 to 2018 and to identify the key characteristics of individuals entitled to utilize this scheme.

The findings of this study may contribute to evaluating whether the subsidy scheme effectively serves socially vulnerable individuals out of labor as intended. Thus, these findings can offer valuable insights for policymakers in assessing resource allocation to ensure a fair, accessible, and inclusive dental care system, ultimately aiming to minimize oral health disparities.

## Methods

### Study design and setting

We used a cohort study design, covering the period from January 1, 2013, to December 31, 2018. The study is situated within Copenhagen municipality, which during the study period, encompassed a population ranging from 549,050 to 622,698, corresponding to approximately 10% of Denmark’s total population.

The foundation of Denmark’s welfare state is the principle that every citizen should have equal access to social and healthcare services. Generally, healthcare services are provided free of charge, with a few exceptions, including dental care for adults. The majority of adults receive dental treatment from private practitioners, with patients bearing roughly 80% of the costs in 2018 [19,20]. To enhance access to dental care for socially vulnerable individuals with low incomes, two distinct schemes are available. The first scheme is the provision of free dental care offered by all municipalities, targeting individuals with severe social and health issues, such as homelessness or substance misuse [21]. This study investigates the second scheme, which is a municipality-administered subsidy scheme, that allows the target group to apply for subsidies for treatments at private dental practices.

#### Public subsidy scheme for dental care services for individuals out of labor

According to the Active Social Policy Act, individuals receiving social benefits equivalent to the cash benefit level are eligible to apply for public subsidies that cover both preventative and curative dental care treatment. To initiate the subsidy application, individuals must initially obtain a cost estimate from a dentist. Depending on the estimate, the application may require pre-approval from the municipality. A law introduced in 2013 states that no pre-approval by the municipality is required if the cost of a treatment course is less than 10,000 DKK (1,344 EUR). However, for treatment courses exceeding this threshold, pre-approval is mandatory before commencing treatment. Applicants are required to submit their applications digitally, along with necessary documentation related to their financial status, including income, expenses, assets, and dental needs. Additionally, applicants are often subject to user payments, which are calculated based on factors such as age, income, and assets. It is also possible to apply for coverage of the user payment [16,17]. As of 2023, the case-processing time is set at 56 days by law.

### Study population

The study population included all adults who met the following criteria: (1) aged 18–65 years, (2) residing in the Copenhagen municipality during the study period, and (3) having at least one registration of receiving out-of-labor social benefits in The Danish Register for Evaluation of Marginalization (DREAM) [22] throughout the study period (Appendix A). Individuals receiving out-of-labor social benefits are typically characterized by having complex social and/or health problems in addition to their unemployed status.

### Outcomes

Data on the subsidy scheme were obtained from the Copenhagen municipality, which provided information on the date of the subsidy decision (granted or rejected) and the amount of the granted subsidy. Three analyses were applied to evaluate the utilization of the public subsidy. First, we examined whether the target population applied for the public subsidy. Secondly, we examined if the subsidy was granted to those who applied. Finally, we examined if the granted subsidy was used among those who received a grant.

For the first two outcomes (applying for a subsidy and receiving a grant), all individuals in the study population were included. For the last outcome (used subsidy), our analysis was restricted to individuals who had received a grant equal to or above 10,000 DKK (1,344 EUR) due to the requirement for pre-approval. Information on used subsidies was obtained from the Danish National Health Service Register [23]. We defined ‘used subsidy’ as the registration of a minimum of four treatments within 12 months of the index date. The unique personal identification number assigned to all residents of Denmark [24], was used to link data from the administrative registers with municipality data.

### Demographic, socioeconomic, and health-related characteristics

Individuals eligible to apply for the subsidy were characterized by registers at Statistics Denmark and the Danish Health Data Authority. For applicants, the index date was the date of the subsidy decision, while for non-applicants, it was set as six months following the first registration of an out-of-labor social benefit during the study period. This timeframe was inserted to ensure individuals have sufficient time to learn about the scheme and apply.

Information regarding demographic characteristics was obtained from the Danish Civil Registration System [24] including gender, age (18–29 years, 30–39, 40–49, 50–65), ethnicity (ethnic Danes, immigrants, descendants), number of years with residence in Denmark (0–3 years, 4–9, 10 or more), and family structure (single with/without children at home, couple with/without children at home).

Information regarding socioeconomic characteristics included the highest level of education attained obtained from the Population’s Education Register [25]. This information was categorized in alignment with the International Standard Classification of Education (ISCED) into short (less than 10 years, ISCED 0–2), medium (10–12 years, ISCED 3–4) and long education (more than 12 years, ISCED 5–8). Information regarding yearly equivalized disposable income at a family level was obtained from the Danish Register on Personal Income and Transfer Payments [26] and categorized into median and quartiles. Historical use of social benefits was obtained from the DREAM register, which provides information on all paid social benefits every week. Historical use of social benefits was based on the duration of receiving out-of-labor social benefits non-stop before the index date, defined as <1 year, 1.0–3.9, and 4.0–5.0 years, respectively. If an individual did not have continuous receipt of out-of-labor social benefits, this was recorded as ‘No.’

The degree of social vulnerability was examined up to 5 years before the index date, using data from the Danish National Patient Register [27], the Danish Psychiatric Central Research Register [28], the Danish National Prescription Registry [29], the National Registry of Alcohol Treatment [30] the Registry of Drug Abusers Undergoing Treatment [31], the Danish Homeless register [32], and the Danish Central Criminal Register [33]. Six indicators of social vulnerability were applied (for further details, refer to Appendix A) [34]: (1) mental illness, (2) alcohol misuse, (3) drug misuse, (4) homelessness, (5) imprisonment, and (6) chronic disease related to substance misuse. Individuals were categorized as having ‘none’, ‘single’, or ‘combined indicators’ defined as having two or more of the ‘single’ indicators.

Information on the historical use of the dental care system (up to three years prior to the index date) was extracted from the Danish National Health Service Register [23]. These data were categorized as (1) no/sporadic use (maximum one filling and/or extraction and/or acute endodontic treatment, and/or root cleaning), (2) irregular use (treatments beyond sporadic use or maximum one examination), or (3) regularly use (minimum two examinations within three years). A wash-out period of 6 months before the index date was inserted to ensure that treatments related to a subsidy grant were not included in the analysis. The use of dental care services after the index date (yes/no) was defined based on whether there was at least one contact within 18 months following the index date. For further details, refer to Appendix A.

### Statistical analyses

At the point of index date, we performed descriptive statistics. Categorical variables were presented as frequencies (N, %), while continuous variables were presented as either the mean and standard deviation (SD) or the median and inter-quartile range (Q1–Q3). To examine the association between demographic, socioeconomic, and health-related characteristics and the utilization of the public subsidy scheme for dental care services, we employed logistic regression analysis. The associations are presented as odds ratio (OR) with 95% confidence interval (CI). The relative concentration index [35] was used to quantify the degree of social inequalities when applying for public subsidies for dental care services. The index has a range of values from −1 to 1 with zero signifying no inequality. A larger value (positive or negative) indicates greater inequality. A negative value represents a higher concentration of the socioeconomic-related variable of concern among the most disadvantaged individuals, while a positive value signifies a higher concentration among the less disadvantaged individuals. In this study, the following socioeconomic variables were included once categorized: highest attained education, household income, historical use of social benefits, and the number of indicators of social vulnerability. The analyses were adjusted for gender and age.

All analyses were performed by SAS software 9.4 and RStudio.

### Ethics

This study was approved by the Danish Data Protection Agency (514-0582/20-3000) and the local Research Ethics Committee at the Faculty of Health and Medical Sciences (504-0334/22-5000). Data were pseudonymized and securely stored within the servers at Statistics Denmark. Informed consent from the subjects included is not required for register-based studies in Denmark when the purpose is solely to perform scientific research.

## Results

**Figure 1:**
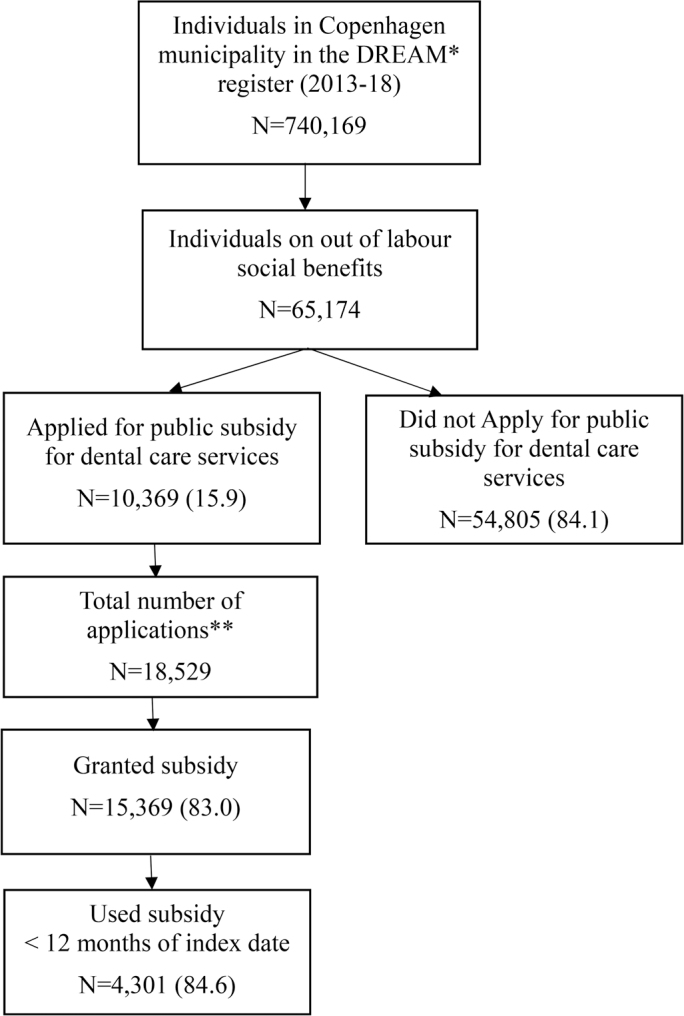
Flow chart of individuals included in the study population from January 1, 2013, to December 31, 2018, stratified into individuals who applied for a subsidy, those whose applications were granted a subsidy, and those who used the granted subsidy within 12 months. The use of subsidies was analyzed for individuals who received a grant equal to or above 10,000 DKK/1,344 EUR (*n* = 5,214). *The Danish Register for Evaluation of Marginalization **Among the total number of applicants, 4,146 (40.0%) individuals were registered withtwo or more applications during the study period

Overall, 65,174 individuals out of labor residing in Copenhagen municipality were eligible to apply for public subsidies for dental care services between 2013-18. Among them, 10,369 individuals (15.9%) applied. The proportion of applicants varied over the years, with an increase from 14.1% in 2013 to 22.8% in 2015, followed by a decline to 14.2% in 2018. In total, 18,529 applications were evaluated, and 15,369 (83.0%) were granted a subsidy. It was observed that 4,146 applicants (40.0%) submitted multiple applications for subsidy during the study period. On average, the granted subsidy amounted to 9,724 DKK/1,303 EUR (SD: 9,923). Among those who received grants equal to or exceeding 10,000 DKK/1,344 EUR (*n* = 5,214, 34.2%), 4,301 individuals (84.6%) used their subsidy within 12 months of the index date (Figure 1.).

### Characteristics of the study population

**Figure 2:**
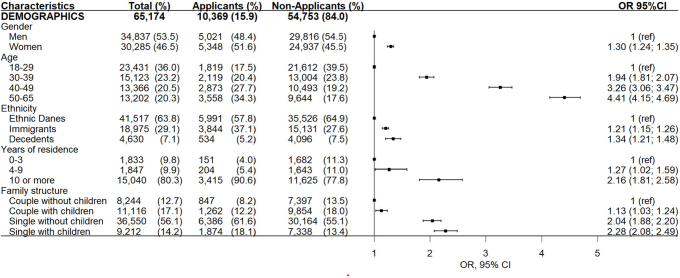
Demographic characteristics of individuals entitled to apply for public subsidies for dental care services in Copenhagen municipality (2013–2018) stratified into applicants and non-applicants. Odds ratio (or) and (95% confidence interval (95% CI) for applying for subsidy adjusted for age and gender. *Among the non-applicants, 52 had missing data due to death or emigration and these were excluded from analyses.

**Figure 3:**
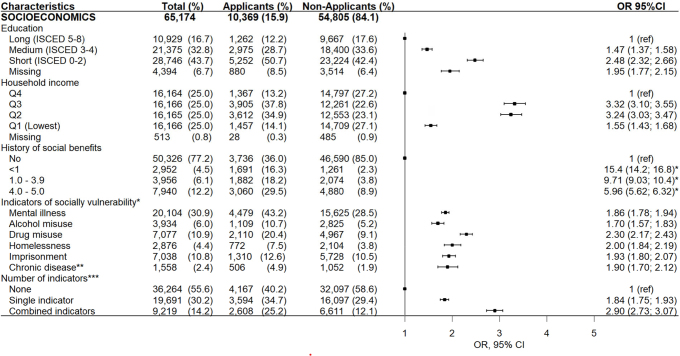
Sociodemographic characteristics of individuals entitled to apply for public subsidies for dental care services in Copenhagen municipality (2013–2018) stratified into applicants and non-applicants. Odds ratio (or) and (95% confidence interval (95% CI) for applying for subsidy adjusted for age and gender. *The effect of historic public support on applying for public subsidy was not shown in the forest plot as it was noticeably larger than the other estimates and expanded the scale. **Reference group = no ***Chronic disease related to substance misuse ****Calculated from the six indicators of social vulnerability

**Figure 4:**
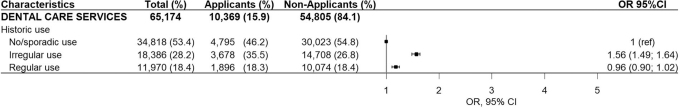
Use of dental care services among individuals entitled to apply for public subsidies for dental care services in Copenhagen municipality (2013–2018) stratified into applicants and non-applicants. Odds ratio (or) and (95% confidence interval (95% CI) for applying for subsidy adjusted for age and gender.

Characteristics of the study population are shown in Figures 2–4. The average age of the total study population was 37.1 (SD: 12.7), with a slightly higher representation of men (53.5%). Most were ethnic Danes (69.8%), single (70.3%), and had a limited prior history of receiving social benefits (77.2%). Short education was registered in 43.7% individuals, and the median annual household income was 120,992 DKK (inter-quartile range:89,802;152,488). Nearly half of the study population exhibited more complex vulnerability demonstrated with either ‘single’ (30.2%) or ‘combined’ (14.2%) indicators. Eight out of 10 had either ‘no/sporadic use’ (53.4%) or ‘irregular use’ (28.0%) of the dental care system before the index date, whereas 55.1% had no contact with the dental care system within 18 months after the index date.

### Characteristics associated with applying for a subsidy

**Figure 5:**
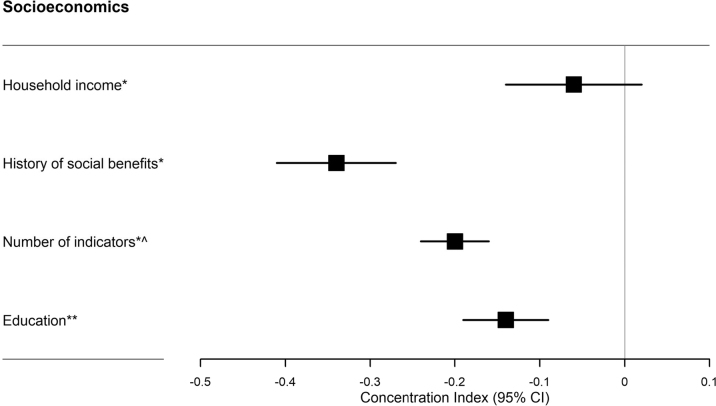
Concentration index [-1;1] of socioeconomic inequalities in applying for public subsidies for dental care services. Concentration index and (95% confidence interval (95% CI). *This analysis was based on 65,122 individuals. ^^^The number of indicators included six indicators of social vulnerability. **This analysis was based on 60,780 individuals as individuals with missing education were not included

The odds for applying for subsidy for dental care services among eligible individuals increased significantly with age, immigrant or descendant status, residence in Denmark for 4 or more years (among immigrants), single marital status, low educational attainment, lower income, a history of receiving social benefits non-stop within the first year or more, exposure to single or combined indicators of social vulnerability, and irregular historical use of dental care services (Figures 2–4). It was observed that individuals who applied for subsidies multiple times during the study period were more likely to be exposed to combined indicators of social vulnerability compared to those who applied only once (see Appendix B). As shown in Figure 5., a history of receiving social benefits emerged as the most pronounced contributing factor to social inequalities when applying for subsidies. The negative value indicated that the history of social benefits was more concentrated among the most disadvantaged individuals.

### Characteristics associated with granted subsidy and used subsidy

The odds of being granted a subsidy among individuals who applied increased significantly with age, single marital status, low educational attainment, income corresponding to Q2–Q3, a history of receiving social benefits non-stop within the first year or more, exposure to combined indicators of social vulnerability, particularly related to alcohol or drug misuse, and homelessness. The odds of using the granted subsidy decreased significantly if the individual was registered as being single or had a history of imprisonment (Appendix B).

## Discussion

This study highlights that only a minority of individuals receiving out-of-labor social benefits in Copenhagen municipality apply for public subsidies for dental care services. Nevertheless, among those who do apply, a large percentage is granted the subsidy, and a large percentage uses it. Applicants, when compared to non-applicants, were more frequently associated with complex social and health problems including a history of receiving social benefits, lower educational attainment, single marital status, and various single and combined indicators of social vulnerability. However, among the large group of non-applicants, a significant proportion of individuals shared the same risk factors for poor oral health, potentially indicating an underserved population that could benefit from the scheme.

Several factors may contribute to the low uptake of the scheme. Firstly, public awareness of the scheme is probably limited. Most of the non-applicants had received social benefits for a shorter period, and some may not have been adequately informed about the scheme. We found that the main driver of social inequality in applying for public subsidies for dental care services is having a history of receiving social benefits. This emphasizes the important role of municipal social workers in identifying individuals with poor oral health as they enter the welfare system and facilitating their access to dental care before the need for large catastrophic treatment arises. Secondly, the requirement of a user fee, even if a subsidy is granted [16,17], can present a substantial challenge for individuals with low income. This is further reinforced as dental care may be perceived as a low priority among vulnerable groups [12]. Thirdly, multiple individual barriers prevent socially vulnerable individuals from accessing oral health care, such as dental fear/anxiety, inadequate oral health literacy, perceived negative attitudes from dental professionals, and communication or language difficulties [12]. Yet, the current subsidy scheme does not address these specific barriers. Finally, the scheme also entails barriers related to the application process. These encompass a high documentation requirement, lengthy case processing time, and potential difficulties in understanding the rules and regulations for applying. The application process places demand on the applicant’s digital skills and oral health literacy, despite the known association between low oral health literacy and poor oral health outcomes [36]. The fluctuations in the scheme’s uptake over the years may be attributed to increasingly stringent requirements over time.

It is a positive finding that individuals facing complex social and health problems are utilizing the scheme, both in terms of applying for and receiving a grant, as these individuals are at higher risk of experiencing poor oral health and, consequently, a greater need for treatment. Social and health-related factors, such as low levels of education, single marital status, immigrant backgrounds, mental illness, and substance misuse, have established associations with poor oral health [37, 38]. This may explain the relatively higher proportion of vulnerable individuals in the applicant group compared to the non-applicant group. Another explanation could be that the most vulnerable residents of Copenhagen municipality can receive social mentoring support and guidance administered by the municipality. This assistance can likely positively influence their ability to apply for public subsidies for dental care services. However, it is important to note that more than 70% of the most vulnerable individuals in our population did not utilize the scheme. This is unfortunate, as their quality of life could be adversely impacted by poor oral health, and improving their access to dental care services could potentially enhance their labor market attachment [39].

It is noteworthy that 15% of individuals fail to use their granted subsidies, even after completing the comprehensive application process. Apart from the applicant’s vulnerability, a potential explanation could lie in the finding that being single was associated with significantly reduced odds of using the grant. Being single may imply a lack of a social support system, which could play a pivotal role in encouraging and facilitating the use of the granted subsidy for accessing dental care services.

Obtaining information on used subsidies was confined to a specific subpopulation, potentially introducing a selection bias that might overestimate our findings. The risk of the grant not being used is higher among individuals who are granted a subsidy exceeding 10,000 DKK (1,344 EUR), as they are required to await pre-approval before treatment can be initiated. Nevertheless, the fact that a portion of individuals do not use their granted subsidy underscores the need for increased awareness and targeted support to ensure that these vulnerable individuals receive the dental care for which they have been granted a public subsidy.

Similar subsidy programs targeting vulnerable groups exist in the Nordic countries, however, they are inadequately described, and there is a lack of comprehensive evaluation [21]. To the best of our knowledge, this public subsidy scheme is not implemented in other settings.

The underlying mechanism explaining the low uptake of the scheme is beyond the scope of this study. Therefore, studies are warranted to explore the experiences of using the dental care system from the vulnerable individual’s perspective, encompassing both the use and non-use of the public subsidy scheme.

The strength of this study includes a large study population and the use of data from high-quality registers with minimal loss to follow-up. The use of several different health and socioeconomic registers enabled a detailed characterization of the study population. The risk for selection bias is limited since the study encompasses all residents of Copenhagen municipality. This study also has some limitations. Firstly, our data lacks information on the oral health status of individuals. Therefore, it is uncertain whether non-applicants needed dental treatment. Yet, it is noteworthy that fewer than one in five individuals in the non-applicant group had been regular attendees of the dental care services before entering the study. Additionally, over half of them had no contact with dental services 18 months after the index date. This, in conjunction with the fact that the non-applicants were unemployed with low incomes, implies that a significant portion of this group likely needed dental care [5,10,40]. Furthermore, our data lacks information on the reasons for rejections and the factors that influence the non-utilization of the subsidy once it has been granted. Lastly, it is important to exercise caution when generalizing these findings, as dental care settings can vary nationally. Nevertheless, The Danish Dental Association reported a national utilization rate of 14.0% for the public subsidy scheme in 2017, which aligns with our findings and enhances the external validity of this study [18].

The public subsidy scheme for dental care services has not undergone significant changes since 2018. Therefore, it is reasonable to assume that the uptake of the scheme remains low today.

## Conclusion

This study highlights a low utilization rate of the public subsidy scheme for dental care services among socially vulnerable individuals out of labor in Copenhagen municipality from 2013 to 2018. Yet, once the individual applies for subsidies, the likelihood of receiving a grant and using it is high. The applicants are characterized by a high degree of vulnerability and a history of limited use of dental care services. However, there is still a substantial portion of individuals out of labor with a potentially high risk of poor oral health who could benefit from using the public subsidy scheme. Given these findings, municipal social workers should pay special attention to recently enrolled welfare recipients and assist them in accessing dental care services through the public subsidy scheme. Furthermore, these findings emphasize the need for policymakers to consider the need for reorganizing the public subsidy scheme in its current form. This may involve reducing organizational barriers, such as eliminating user payments and simplifying the application process. Additionally, it may involve addressing individual barriers to accessing dental care services to enhance the uptake of the public subsidy scheme. Finally, implementing oral health interventions targeting socially vulnerable individuals out of labor can significantly enhance their access to dental care services.

## Supplementary Material

Utilization of a public subsidy scheme for dental care services among socially vulnerable citizens out of labor in Copenhagen, Denmark

## Data Availability

Due to data privacy regulations by Statistics Denmark, the data generated during this study are not publicly accessible.
