# Supplementary material for: Utilization of a public subsidy scheme for dental care services among socially vulnerable citizens out of labor in Copenhagen, Denmark
Source: Acta Odontol Scand. 2024 Mar 26;83:40289. doi: 10.1080/00016357.2023.2279606 (PMC11302627; doi:10.1080/00016357.2023.2279606)
Supplement: Utilization of a public subsidy scheme for dental care services among socially vulnerable citizens out of labor in Copenhagen, Denmark [file AOS-83-40289-s1.pdf]

Supplementary material has been published as submitted. It has not been copyedited or typeset by Acta Odontologica Scandinavica.

Appendix A

Table A Out-of-labor social benefits codes extracted from the Danish Register for Evaluation of Marginalization (DREAM) for inclusion

**Type of out-of-labor social benefit**	**Code**
Cash-benefit	130,133-139, 710,713-719, 730,733-739
Educational benefit	140,143-149, 720,723-729
Integration benefit	160,163-169, 700,703-709
Availability benefit	740,743-749
Rehabilitation benefit	750,753-758,760,763-768
Resource course grant	810,813-818
Job clarification	870,873-878

Table B Indicators of social vulnerability and operationalization including source of information

**Indicator**	**Operationalization and source of information**
Mental illness	a) at least one hospital contact according to the International Classification of Disease 10th revision (ICD-10: F321, F322, F328, F329, F331, F332, F334-F339, F603, F20-F31, F34-F43, F50, F60-F62, F90-F92) (24)(25) *or*<br>b) having at least 5 prescription redemptions for mental disease in the Anatomical Therapeutic Chemical (ATC) Classification System: N06A, N05B, N06BA04, N06BA09, N06Ba12, N05X12, N05X13, N05AH03, N05AX08, N05AX09, N03AG01, N05AH04, N05BA (excluding N06AA09, N05BA02) (26)
Alcohol misuse	a) at least one hospital contact (ICD-10: F101-F109) (24) *or*<br>b) at least one redeemed prescription redemption for alcohol addiction (ATC: N07BB) (26) *or*<br>c) minimum one registration at a national alcohol treatment center (27) *or*<br>d) minimum one registration with a daily alcohol intake at a national drug treatment center (28)
Drug misuse	a) at least one hospital contact (ICD-10: F111-114, F119, T401, T403, F141-F143, F149 F151-F153, F159, F161-F162, F169, F191-F194, F199, F121-F122, F129, F131-F134, F139 F181-F182, F189) (24) *or*<br>b) at least one redeemed prescription for drug addiction (ATC: N07BC, N05BA) (26) *or*

	c) minimum one registration at a drug treatment center (28) *or*<br>d) a registration at a national alcohol treatment center within the last month of a minimum of one specified drug: heroin, methadone, opiate, barbiturate, cocaine, amphetamine, hallucinogens, inhaler, or others (27)
Homelessness	Minimum one contact with a public homeless shelter (29)
Unconditional imprisonment	Any unconditional imprisonment sentence excluding sentences for traffic offenses (1460, 2110, 2210, 2220, 2420, 2610, 3835) (30)
Substance misuse-related illness	At least one hospital contact (ICD-10: B18, B20-B24, G62, K70-K77, K86) (24)

Table C Codes were extracted from the Danish National Health Service Register (20) and operationalized to assess the historical use of the dental care system

**Variable**	**Operationalization**
No/sporadic use	a) no registration *or* maximum:<br>b) one filling (1500-1507, 1509,1550-1559, 4510-4515, 5420-4525) *and/or*<br>c) one extraction (1700-1703, 1801) *and/or*<br>d) one acute endodontic treatment (1600, 1601, 1605) *and/or*<br>e) one root cleaning (1431)
Irregularly use	Treatments beyond sporadic use *or* a maximum of one examination<br><br>Treatments:<br>1300-1302, 1310-1410, 1420, 1425, 1430, 1431, 1440, 1452-1454, 1500-1507, 1509, 1550-59, 1600-06, 1700-03, 1800, 1801, 2910, 2920, 4510-15, 4520, 4521-25, 492921<br><br>Examinations:<br>1001, 1100, 1110-1116, 1120, 1130, 1140-41, 1160, 1170,1171, 1180, 1415, 2110, 2120, 491000-491115, 491140, 491141, 491181
Regular use	A minimum of 2 examinations (codes extracted from above)

Appendix B

Table 1a Demographic characteristics of total number of applications for public subsidies for dental care services in Copenhagen municipality (2013-2018), stratified by applications being granted versus not granted. The odds ratio (95% confidence interval) for being granted adjusted for age and gender.

	**Total no of applications** 18,529	**Granted** 15,369 (83.0)	**Not granted** 3,160 (17.0)	**OR** (95% CI)
Gender				
Male	8,642 (46.6)	7,166 (46.6)	1,476 (46.7)	1
Women (n, %)	9,887 (53.4)	8,203 (53.4)	1,684 (53.3)	1.00 (0.93-1.08)
Age				
Age (mean, SD)	44.5 (11.6)	44.6 (11.5)	43.7 (11.9)	
18-29	2,676 (14.4)	2,162 (14.1)	514 (16.3)	1
30-39	3,354 (18.1)	2,751 (17.9)	603 (19.1)	1.09 (0.95-1.24)
40-49	5,293 (28.6)	4,417 (28.7)	876 (27.7)	1.20 (1.06-1.35)
50-65	7,206 (38.9)	6,039 (39.3)	1,167 (36.9)	1.23 (1.10-1.38)
Ethnicity				
Ethnic Danes	10,838 (58.5)	8,978 (58.4)	1,860 (58.9)	1
Immigrants	6,857 (37.0)	5,734 (37.3)	1,123 (35.5)	1.04 (0.96-1.13)
Decedents	834 (4.5)	657 (4.3)	177 (5.6)	0.83 (0.69-0.99)
Years with residence in Denmark				
0-3	197 (2.9)	164 (2.9)	33 (3.0)	1
4-9	301 (4.5)	247 (4.4)	54 (4.9)	0.90 (0.56-1.45)
10 or more	6,216 (92.8)	5,207 (92.7)	1,009 (92.1)	1.00 (0.68-1.49)
Family structure				
Couple without children at home	1,316 (7.1)	1,053 (6.9)	263 (8.3)	1
Couple with children at home	1,904 (10.3)	1,523 (9.9)	381 (12.1)	1.00 (0.84-1.20)
Single with children at home	3,370 (18.2)	2,842 (18.5)	528 (16.7)	1.34 (1.14-1.59)
Single without children at home	11,939 (64.4)	9,951 (64.7)	1,988 (62.9)	1.24 (1.08-1.44)

Table 1b Sociodemographic characteristics of total number of applications for public subsidies for dental care services in Copenhagen municipality (2013-2018), stratified by applications being granted versus not granted. The odds ratio (95% confidence interval) for being granted adjusted for age and gender.

	**Total no of applications** 18,529	**Granted** 15,369 (83.0)	**Not granted** 3,160 (17.0)	**OR** (95% CI)
Education				
Long (>12 years)	2,344 (12.7)	1,903 (12.4)	441 (14.0)	1
Medium (9-12 years)	5,396 (29.1)	4,447 (28.9)	949 (30.0)	1.10 (0.97-1.25)
Short (<9 years)	9,245 (49.9)	7,716 (50.2)	1,529 (48.4)	1.24 (1.10-1.40)
Missing	1,544 (8.3)	1,303 (8.5)	241 (7.6)	1.27 (1.07-1.51)
Income				
Median annual income	124,507	124,431	124,757	
Q1;Q3	105,642;139,671	105,976;139,049	103,489;143,651	-
Q1 (lowest)	4,696 (25.3)	3,813 (24.8)	883 (27.9)	1.07 (0.96-1.19)
Q2	4,585 (24.7)	3,883 (25.3)	702 (22.2)	1.33 (1.19-1.48)
Q3	4,581 (24.7)	3,912 (25.5)	669 (21.2)	1.40 (1.25-1.56)
Q4	4,667 (25.2)	3,761 (24.5)	906 (28.7)	1
Historic public support				
No	5,170 (27.9)	3,988 (26.0)	1,182 (37.4)	1
<1 year	2,700 (14.6)	2,254 (14.7)	446 (14.1)	1.50 (1.33-1.69)
1.0-3.9	3,165 (17.1)	2,639 (17.2)	526 (16.7)	1.49 (1.33-1.67)
4.0-5.0	7,494 (40.4)	6,488 (42.2)	1,006 (31.8)	1.92 (1.74-2.11)
Indicators of social vulnerability (ref=none)				
Mental illness	8,643 (46.6)	7,174 (46.7)	1,469 (46.5)	1.01 (0.94-1.09)
Alcohol misuse	2,100 (11.3)	1,787 (11.6)	313 (9.9)	1.17 (1.03-1.33)
Drug misuse	4,168 (22.5)	3,547 (23.1)	621 (19.7)	1.22 (1.11-1.34)
Homelessness	1,351 (7.3)	1,155 (7.5)	196 (6.2)	1.24 (1.06-1.46)
Imprisonment	2,204 (11.9)	1,793 (11.7)	411 (13.0)	0.91 (0.81-1.03)
Chronic disease	1,092 (5.9)	930 (6.1)	162 (5.1)	1.16 (0.97-1.38)
Number of indicators				
Single indicator	6,470 (34.9)	5,326 (34.6)	1,144 (36.2)	1.00 (0.92-1.10)
Combined indicators	5,092 (27.5)	4,295 (28.0)	797 (25.2)	1.15 (1.04-1.27)
None	6,967 (37.6)	5,748 (37.4)	1,219 (38.6)	1

* Chronic disease related to substance misuse

Table 1c Characteristics of the dental care system utilization: total number of applications for public subsidies for dental care services in Copenhagen municipality (2013-2018), stratified by applications being granted versus not granted. The odds ratio (95% confidence interval) for being granted adjusted for age and gender.

	**Total no of applications** 18,529	**Granted** 15,369 (83.0)	**Not granted** 3,160 (17.0)	**OR** (95% CI)
Historic use of dental care services				
No/sporadic use	6,152 (33.2)	5,075 (33.0)	1,077 (34.1)	1
Irregular use	7,237 (38.1)	6,004 (39.1)	1,233 (39.0)	1.03 (0.95-1.13)
Regular use	5,140 (27.7)	4,290 (27.9)	850 (26.9)	1.05 (0.95-1.16)
Use of dental care services 18 months after the index date				
Contact	16,033 (86.5)	13,767 (89.6)	2,266 (71.7)	
No contact	2,496 (13.5)	1,602 (10.4)	894 (28.3)	

Table 2a Demographic characteristics of the total number of applications that received grants equal to or above 10,000 DKK (1,344 EUR) for dental care services in Copenhagen municipality (2013-2018), stratified by grants used versus not used within 12 months of the index date. The odds ratio (95% confidence interval) for grant utilization, adjusted for age and gender.

	**Total**<br>5,083	**Grant used**<br>4,301 (84.6)	**Grant not used**<br>782 (15.4)	**OR**<br>(95% CI)
Gender				
Male	2,753 (54.2)	2,403 (54.3)	350 (53.2)	1
Women (n, %)	2,330 (45.8)	2,022 (45.7)	308 (46.8)	0.96 (0.81-1.13)
Age				
Age (mean, SD)	44.6 (11.2)	44.4 (11.3)	45.6 (10.9)	
18-29	673 (13.2)	598 (13.5)	75 (11.4)	1
30-39	959 (18.9)	848 (19.2)	111 (16.9)	0.96 (0.70-1.31)
40-49	1,509 (29.7)	1,310 (29.6)	199 (30.2)	0.83 (0.62-1.10)
50-65	1,942 (38.2)	1,669 (37.7)	273 (41.5)	0.77 (0.59-1.01)
Ethnicity				
Ethnic Danes	3,018 (59.4)	2,647 (59.8)	371 (56.4)	1
Immigrants	1,815 (35.7)	1,559 (35.2)	256 (38.9)	0.88 (0.74-1.04)
Decedents	250 (4.9)	219 (4.6)	31 (4.7)	0.87 (0.58-1.32)
Years with residence in Denmark (among immigrants)				
0-3*	69 (3.9)	N/A*	N/A*	N/A*
4-9	80 (4.5)	67 (4.4)	13 (5.2)	N/A*
10 or more	1,637 (91.7)	1,404 (91.4)	233 (93.2)	N/A*
Family structure				
Couple without children at home	365 (7.2)	332 (7.5)	33 (5.0)	1
Couple with children at home	505 (9.9)	441 (10.0)	64 (9.7)	0.67 (0.43-1.05)
Single with children at home	896 (17.6)	774 (17.5)	122 (18.5)	0.63 (0.41-0.95)
Single without children at home	3,317 (65.3)	2,878 (65.0)	439 (66.7)	0.65 (0.45-0.94)

*N/A: The analysis was not applicable due to the limited number of observations

Table 2b Sociodemographic characteristics of the total number of applications that received grants equal to or above 10,000 DKK (1,344 EUR) for dental care services in Copenhagen municipality (2013-2018), stratified by grants used versus not used within 12 months of the index date. The odds ratio (95% confidence interval) for grant utilization, adjusted for age and gender.

	**Total**<br>5,083	**Grant used**<br>4,301 (84.6)	**Grant not used**<br>782 (15.4)	**OR**<br>(95% CI)
Education				
Short (<9 years)	2,702 (53.2)	2,345 (53.0)	357 (54.3)	0.96 (0.72-1.28)
Medium (9-12 years)	1,410 (27.4)	1,240 (28.0)	170 (25.8)	1.13 (0.83-1.53)
Long (>12 years)	484 (9.5)	418 (9.5)	66 (10.0)	1
Missing	487 (9.6)	422 (9.5)	65 (9.9)	1.00 (0.69-1.45)
Income				
Median annual income	122,963	122,817	123,359	-
Q1;Q3	104,040;137,218	104,040;137,108	104,093;138,156	-
Q1 (lowest)	1,281 (25.2)	1,105 (25.0)	176 (26.8)	0.92 (0.73-1.16)
Q2	1,267 (24.9)	1,118 (25.3)	149 (22.6)	1.17 (0.92-1.48)
Q3	1,260 (24.8)	1,097 (24.8)	163 (24.8)	1.05 (0.84-1.33)
Q4	1,275 (25.1)	1,105 (25.0)	170 (25.8)	1
Missing				
Historic public support				
No	1,388 (27.3)	1,212 (27.4)	176 (26.8)	1
<1 year	781 (15.4)	673(15.2)	108 (16.4)	0.93 (0.72-1.20)
1.0-3.9	838 (16.5)	748 (16.9)	90 (13.7)	1.26 (0.96-1.66)
4.0-5.0	2,076 (41.8)	1,792 (40.5)	284 (43.2)	0.98 (0.80-1.21)
Indicators of social vulnerability (ref=none)				
Mental illness	2,222 (43.7)	1,955 (44.2)	267 (40.6)	1.17 (0.99-1.38)
Alcohol misuse	704 (13.9)	615 (13.9)	89 (13.7)	1.07 (0.84-1.36)
Drug misuse	1,348 (26.5)	1,166 (26.4)	182 (27.7)	0.94 (0.78-1.13)
Homelessness	506 (10.0)	428 (9.7)	78 (11.9)	0.77 (0.60-1.00)
Imprisonment	761 (15.0)	629 (14.2)	132 (20.1)	0.58 (0.47-0.73)
Chronic disease*	356 (7.0)	300 (6.8)	56 (8.5)	0.81 (0.60-1.09)

Number of indicators				
Single indicators	1,672 (32.9)	1,472 (33.3)	200 (30.4)	1.09 (0.89-1.34)
Combined indicators	1,592 (31.3)	1,373 (31.0)	219 (33.3)	0.94 (0.77-1.15)
None	1,819 (35.8)	1,580 (35.7)	239 (36.2)	1

*Chronic disease related to substance misuse

Table 2c Characteristics of the dental care system utilization: total number of applications that received grants equal to or above 10,000 DKK (1,344 EUR) for dental care services in Copenhagen municipality (2013-2018), stratified by grants used versus not used within 12 months of the index date. The odds ratio (95% confidence interval) for grants utilization, was adjusted for age and gender.

	**Total** 5,083	**Grant used** 4,301 (84.6)	**Grant not used** 782 (15.4)	**OR** (95% CI)
Historic use of dental care services				
No/sporadic use	2,238 (44.0)	1,968 (44.5)	270 (41.0)	1
Irregular use	2,095 (41.2)	1,805 (40.8)	290 (44.1)	0.85 (0.71-1.02)
Regular use	750 (14.8)	652 (14.7)	98 (14.9)	0.94 (0.73-1.21)
Use of dental care services 18 months after the index date				
Contact	4,829 (95.0)	4,425 (100)	404 (61.4)	-
No contact	254 (5.0)		254 (38.6)	-

Table 3 Number of individuals who applied for public subsidies for dental care services once versus multiple times during the study period in Copenhagen municipality (2013-2018), stratified by the number of indicators of social vulnerability (none, single or combined indicators).

	**Indicators of social vulnerability*** (n, %)			
**Applied for public subsidy** (n, %)				
	None	Single	Combined	Total
Once	2,641 (42.4)	2,144 (34.5)	1,438 (23.1)	6,233 (60.0)
Multiple times	1,526 (36.8)	1,450 (35.0)	1,170 (28.2)	4,146 (40.0)
Total	4,167 (40.2)	3,594 (34.7)	2,608 (25.2)	10,369 (100)

*An individual was classified as having 'none', ‘single’, or ‘combined indicators’ defined by having two or more of the ‘single’ indicators: 1) mental illness, 2) alcohol misuse, 3) drug misuse, 4) homelessness, 5) imprisonment, and 6) chronic disease related to substance misuse
